# A25 INVESTIGATING THE ROLE OF TOLL-INTERACTING PROTEIN DURING *CITROBACTER RODENTIUM* INFECTION

**DOI:** 10.1093/jcag/gwae059.025

**Published:** 2025-02-10

**Authors:** P Forneris, R Hannawayya, K Cirone, E Cobo

**Affiliations:** University of Calgary Cumming School of Medicine, Calgary, AB, Canada; University of Calgary, Faculty of Veterinary Medicine, Calgary, AB, Canada; University of Calgary, Faculty of Veterinary Medicine, Calgary, AB, Canada; University of Calgary, Faculty of Veterinary Medicine, Calgary, AB, Canada

## Abstract

**Background:**

Diarrheal diseases are a threat to human health, being the second leading cause of death in children. Diarrheic enterocolitis can be caused by attaching/effacing (A/E) pathogens like enteropathogenic and enterohemorrhagic *Escherichia coli* (EPEC and EHEC). *Citrobacter rodentium* (CR), a mouse A/E pathogen, mimics EPEC and EHEC infections, causing goblet cell depletion, crypt hyperplasia, and leukocyte infiltration. Autophagy, a critical immune pathway linked to goblet cell mucus secretion, is involved in the defence against CR. Toll-interacting protein (Tollip) is an intracellular mediator of autophagy and immunity, but its role in A/E infections remains elusive. This study aims to test the hypothesis that Tollip plays a protective role in CR infection.

**Aims:**

- Assess CR infection kinetics in WT and *Tollip*^-/-^ mice.

- Assess colitis in CR-infected WT and *Tollip*^-/-^ mice.

**Methods:**

Bacterial load was assessed by fecal colony forming unit (CFU) count in CR-infected WT and *Tollip*^-/-^ mice. Histological damage was evaluated by scoring epithelial damage, leukocyte infiltration and crypt hyperplasia in H&E-stained distal colon sections. Apoptotic cells were identified by TUNEL. Acid and neutral mucin production was evaluated by Alcian-PAS staining. Glycosylation patterns were determined by immunofluorescence with lectins specific to sialic acid- and fucose-bound mucins.

**Results:**

*Tollip* ^-/-^ and WT mice showed similar infection kinetics during the establishment and expansion phases (2-6 days post-infection (dpi)) and steady-state (7-14 dpi), with a peak of bacterial counts at 10 dpi. WT mice cleared CR by day 15, but *Tollip*^-/-^ still shed bacteria by 17 dpi. The colitis grade was similar between WT and *Tollip*^-/-^ mice, with marked epithelial damage at 8 dpi and increased leukocyte infiltration at 17 dpi. Scarce apoptotic cells (<4 per field) were detected in WT and *Tollip*^-/-^ mice during infection. *Tollip*^-/-^ showed a thinner colonic mucin barrier and less mucin-filled goblet cells at 17 dpi compared to WT mice. CR-infected *Tollip*^-/-^ mice showed altered mucin glycosylation patterns, with lower fucosylated mucins in the upper crypt and lumen and increased sialic acid-bound mucins in the luminal barrier and lower crypt. The mucin barrier, goblet cells, and mucin glycosylation were similar in uninfected *Tollip*^-/-^ and WT mice.

**Conclusions:**

This study demonstrates a new protective role for Tollip, particularly during the resolution phase, as a lack of Tollip delayed the clearance of CR. This phenotype could be explained by deficiencies in the mucin layer observed in *Tollip*^-/-^ mice, evidenced by lower mucin production and anomalies in mucin glycosylation. By enhancing our understanding of host-pathogen interactions during enteric infections, this research serves as a step in the development of new treatments and prevention strategies.

**Funding Agencies:**

**None**

